# Cdon suppresses vascular smooth muscle calcification via repression of the Wnt/Runx2 Axis

**DOI:** 10.1038/s12276-022-00909-7

**Published:** 2023-01-06

**Authors:** Byeong-Yun Ahn, Yideul Jeong, Sunghee Kim, Yan Zhang, Su Woo Kim, Young-Eun Leem, Jong-Sun Kang

**Affiliations:** 1 Department of Molecular Cell Biology, Single Cell Network Research Center, Sungkyunkwan University, School of Medicine, Suwon, South Korea; 2 Research Institute of Aging Related Disease, AniMusCure, Inc., Suwon, South Korea

**Keywords:** Calcification, Stress signalling

## Abstract

Osteogenic transdifferentiation of vascular smooth muscle cells (VSMCs) is a risk factor associated with vascular diseases. Wnt signaling is one of the major mechanisms implicated in the osteogenic conversion of VSMCs. Since Cdon has a negative effect on Wnt signaling in distinct cellular processes, we sought to investigate the role of Cdon in vascular calcification. The expression of Cdon was significantly downregulated in VSMCs of the aortas of patients with atherosclerosis and aortic stenosis. Consistently, calcification models, including vitamin D3 (VD3)-injected mice and VSMCs cultured with calcifying media, exhibited reduced Cdon expression. Cdon ablation mice (cKO) exhibited exacerbated aortic stiffness and calcification in response to VD3 compared to the controls. Cdon depletion induced the osteogenic conversion of VSMCs accompanied by cellular senescence. The Cdon-deficient aortas showed a significant alteration in gene expression related to cell proliferation and differentiation together with Wnt signaling regulators. Consistently, Cdon depletion or overexpression in VSMCs elevated or attenuated Wnt-reporter activities, respectively. The deletion mutant of the second immunoglobulin domain (Ig2) in the Cdon ectodomain failed to suppress Wnt signaling and osteogenic conversion of VSMCs. Furthermore, treatment with purified recombinant proteins of the entire ectodomain or Ig2 domain of Cdon displayed suppressive effects on Wnt signaling and VSMC calcification. Our results demonstrate a protective role of Cdon in VSMC calcification by suppressing Wnt signaling. The Ig2 domain of Cdon has the potential as a therapeutic tool to prevent vascular calcification.

## Blood vessels: A pathway to prevent calcium build-up

Researchers in South Korea have identified a molecular signaling pathway that suppresses calcium build-up (calcification) of muscle tissue in blood vessels, opening a new avenue for preventing health problems such as atherosclerosis, chronic kidney disease and diabetes. Young-Eun Leem, Jong-Sun Kang and colleagues at Sungkyunkwan University, Suwon, investigated the role of a glycoprotein (a protein-carbohydrate combination) called Cdon, a ‘cell adhesion molecule’ involved in binding cells to other cells. In studies in mice, they identified a sequence of molecular interactions by which Cdon can suppress the calcification of smooth muscle cells, the most abundant cells in artery walls. This calcification, commonly known as hardening of the arteries, can cause heart disease and strokes, among other medical problems. The Ig2 domain of Cdon identified by the researchers may have therapeutic potential.

## Introduction

The contractile properties of vascular smooth muscle cells (VSMCs) are critical for the maintenance of vascular function^1^. VSMCs express a variety of contractile proteins, such as alpha-smooth muscle actin (αSMA), smooth muscle myosin heavy chain (sm-MHC), and smooth muscle 22α (SM22α), ensuring contractile function. In response to diverse physiological and pathological stimuli, VSMCs undergo a process called cellular remodeling^2^. In homeostatic remodeling, VSMCs dedifferentiate to proliferate and redifferentiate into muscle cells to regain their contractile properties. Upon pathological insults, VSMCs undergo a phenotypic switch from the contractile to synthetic phenotype and eventually transdifferentiate into osteochondrogenic cell types characterized by the loss of VSMC marker proteins and gain of osteoblast cell marker proteins (such as Runt-related transcription factor 2 (Runx2) and osteopontin)^3,4^. The resulting cells produce mineralizing matrices, and therefore, the osteochondrogenic transdifferentiation of VSMCs is the key event for vascular calcification associated with diverse pathological conditions, such as aging, diabetes, atherosclerosis, and chronic kidney disease^5–7^. Among multiple signaling pathways, canonical Wnt signaling is involved in osteogenic transdifferentiation and vascular calcification. In VSMCs, Wnt signaling is activated by a high level of phosphate and induces Runx2 expression, a key transcription factor for osteochondrogenic transdifferentiation^8,9^. A recent study demonstrated that the inhibition of Wnt signaling by magnesium reverses the osteogenic conversion of VSMCs^10^. Thus, understanding the regulatory mechanisms of Wnt signaling in VSMCs is important to develop therapeutic strategies against vascular calcification.

Cdon (CAM-related/downregulated by oncogenes) is a member of the immunoglobulin/fibronectin type III superfamily of cell adhesion molecules. Cdon plays critical roles in the development of forebrain and skeletal muscle via regulation of sonic hedgehog (Shh), Wnt, and N-cadherin/cell adhesion signaling^11–13^. A recent study proposed Cdon as a therapeutic target to promote endothelial integrity in response to acute inflammation via inhibition of desert hedgehog-mediated signaling^14^. In addition to Shh signaling activation by Cdon as a coreceptor, Cdon suppresses Wnt signaling by interacting with the LRP6 coreceptor to promote ventral neuronal cell fates in early forebrain development^15^. A similar suppressive effect of Cdon on Wnt signaling has also been demonstrated in the prevention of cardiac remodeling and fibrosis^16^. Considering the importance of the fine control of Wnt signaling in the vascular system, the question arises of whether Cdon plays a role in vascular calcification. The initial open data analysis revealed that Cdon transcripts are decreased in calcified aortas compared to normal aortas. Consistently, Cdon expression showed a negative correlation with osteogenic markers (Runx2, Osterix, and Osteopontin). Therefore, we generated a tamoxifen (tmx)-inducible *SM22α-Cre* ^*ERT2*^*;Cdon* (*Cdon*^*f/f*^) mouse model to ablate Cdon in smooth muscle upon tmx administration. Using these mice, we examined the role of Cdon in vascular calcification induced by vitamin D. Cdon depletion in VSMCs exacerbated VSMC calcification concurrent with enhanced Wnt signaling. Conversely, Cdon overexpression attenuated the osteogenic conversion of VSMCs by suppressing Wnt signaling. The Cdon deletion mutant of the Ig2 domain, which is responsible for Wnt suppressive activity, failed to attenuate the osteogenic conversion of VSMCs. In addition, treatment with purified Ig2-Fc (Ig2 domain fused to Fc domain of human IgG gamma protein) suppressed the calcification of VSMCs. Collectively, these data demonstrate a protective role of Cdon in vascular calcification via suppression of Wnt signaling. Furthermore, the Ig2 domain of Cdon has the potential as a therapeutic agent to intervene in vascular calcification.

## Materials and methods

### Animal studies

Mice bearing the Cdon-floxed allele (*Cdon*^*f/f*^) were obtained from EUCOMM and maintained as previously described^17^. For Cdon deletion in vascular muscle cells, *Cdon*^*f/f*^ mice were crossed with *SM22a-Cre*^*ERT2*^ mice, which express tmx-inducible Cre recombinase under the transcriptional control of the *SM22a* (Tagln, smooth muscle protein 22-alpha) promoter. *SM22α-Cre*^*ERT2*^ mice were obtained from Severance Integrative Research Institute for Cerebral & Cardiovascular Diseases (SIRIC, Yonsei University Health System, Seoul, S. Korea). For deletion of one of the exons of the *Cdon* gene in VSMCs, approximately 8- or 10-week-old mice were injected intraperitoneally with 100 mg/kg tmx (Sigma-Aldrich, St. Louis, USA) every two days five times. For the model of vascular calcification in mice, 8- or 10-week-old C57BL/6 male mice and vehicle- or tmx-injected mice were administered VD3, which induces vascular calcification by accelerating calcium levels in the blood (5 × 10^5^ IU/kg per day; Cayman Chemical, Michigan, USA). VD3 (14.575 mg) in 70 μl of ethanol was mixed with 500 μl of cremophor for 15 min at room temperature, and this solution was then mixed with 6.2 ml of sterilized water, including 250 mg of dextrose for 15 min at room temperature. The mice were injected with a dose of VD3 (150 μl/25 g per day) subcutaneously for 3 consecutive days^18^.

The animal experiments in this study were approved by the Institutional Animal Care and Use Committee (IACUC) of Sungkyunkwan University School of Medicine (SUSM) and complied with the animal experiments guidelines of the SUSM Ethics Committee (protocol number: SKKUIACUC 2020-04-14-1).

### Echocardiography

For measurement of cardiac function and pulse wave velocity (PWV), echocardiographic analysis was performed 1 day before sacrifice. Mice were anesthetized with 1–2% (vol/vol) isoflurane, and body temperature was maintained at 36–38 °C with a heating lamp and a heating platform. Heart rates were monitored and generally maintained at 400–500 beats per minute. Echocardiography was carried out using a Vevo LAZR-X machine (the BIORP of Korea Basic Science Institute) with a 40-MHz probe (visual sonic). Analysis of M-mode images derived from the short-axis view of the left ventricle was carried out to measure the ejection fraction and fractional shortening^19^. PWV was obtained from the B-mode and pulse-waved (PW) Doppler mode of the aortic arch view, calculated as PWV = aortic arch distance/transit time (cm/s). The PW Doppler mode sample volume was placed in the ascending aorta, and the time (T1) from the onset of the QRS complex to the onset of the ascending aortic Doppler waveform was measured. On the same image plane, the PW Doppler mode sample volume was placed as distal as possible in the descending aorta, and the time (T2) from the onset of the QRS complex to the onset of the ascending aortic Doppler waveform was measured. The obtained values for T1 and T2 were averaged over 10 cardiac cycles. The aortic arch distance was measured between the 2 sample volume positions along the central axis of the aortic arch on the B-mode image, and the transit time was calculated by T2 − T1 (ms)^20^.

### Cell culture

A7r5 (ATCC, CRL-1444, Manassas, USA) cells were cultured in a normal medium containing DMEM, 10% FBS, and 1% penicillin‒streptomycin as previously described^21^. For induction of vascular calcification in vitro, the cells incubated in normal media were switched to calcifying medium (CM) that contains excess levels of phosphate and calcium, leading to mineral deposition in cells. CM was generated by adding 100 nM dexamethasone, 1 mM insulin, 50 μg/ml ascorbic acid, 10 mM β-glycerophosphate, and 8 mM CaCl_2_ to normal media^18^. The media were replenished every 48 h for up to 3 days or 7 days, and the first day of culture in CM was defined as Day 0. For overexpression studies, pBABE-puro or pBABE-puro-Cdon was transfected into A7r5 cells by using 1 mg/ml polyethyleneimine (PEI, Sigma-Aldrich, St. Louis, USA). For depletion of Cdon in A7r5 cells, control shRNA (shCont) or Cdon shRNA (shCdon) was expressed by using a lentiviral expression system as previously described^17^. For analysis of the effects of Cdon on Wnt signaling, cells were treated with 20 ng/ml Wnt3a (R&D Systems, Minneapolis, USA) or 5 μM XAV939 (Calbiochem, San Diego, USA).

### Histological analysis

Aortas were isolated from PBS-perfused mice, fixed with 4% PFA, embedded in paraffin, and sectioned at a thickness of 5 μm. For the determination of calcium deposition in the aortas, slide sections were stained with Von Kossa stain. Briefly, deparaffinized sections were incubated with 1% silver nitrate solution under ultraviolet light for 2 h, followed by the removal of unreacted silver with 5% sodium thiosulfate for 5 min. Sections were counterstained with nuclear fast red for 5 min before dehydration and mounting^18^. For quantification of the calcium deposition in aortas, the images obtained from TissueFAXS PLUS (TissueGnostics, Wien, Austria) were analyzed with ImageJ software (NIH, Bethesda, USA).

### Immunofluorescence

For the immunohistochemistry of aortic samples, deparaffinized samples were boiled in Tris-EDTA buffer (pH 9.0, 0.05% Tween-20) for antigen retrieval followed by the standard protocol. For analysis of cell death during vascular calcification, a Click-iT TUNEL assay Alexa imaging assay kit (Invitrogen, C10246, Waltham, USA) was utilized on cryosections of aortas that were isolated from PBS-perfused mice and sectioned at a thickness of 5 μm. Fluorescence images were analyzed with an LSM-710 confocal microscope (Carl Zeiss, Jena, Germany) as previously described^16^.

### Protein and RNA analysis

Immunoblot analysis was carried out as previously described^16^. In brief, cultured cells and homogenized aortic tissues were lysed in RIPA buffer (protease inhibitor cocktail (Roche, 1183617001, Basel, Switzerland), pH 8.0; 150 mM NaCl; 1 mM EDTA; 1% Triton X-100; 10 mM Tris-HCl). The primary antibodies used in this study are listed in Supplementary Table 1.

Quantitative reverse transcription polymerase chain reaction (RT-PCR) and RNA sequencing analysis were performed as previously described^16^. Total RNA from cells or homogenized tissues was extracted with easy-BLUE (iNtRON, Seongnam, S. Korea) reagent following the manufacturer’s instructions. cDNA samples were generated from 0.5 mg of RNA by PrimeScript RT reagent (TaKaRa, San Jose, USA) according to the manuscript’s protocol. The primer sequences used in this study are listed in Supplementary Table 2. High-throughput sequencing was performed as single-end 75 sequencings using Illumina NExtSEq 500 (eBioscience, San Diego, USA). The analysis of RNA sequencing data was performed by using ExDEGA v3.2 (eBioscience, San Diego, USA), and a heatmap was displayed utilizing Morpheous (http://software.broadinstitute.org/morpheus/). Global gene expression was assessed by the Reactome with GSEA (http://www.gsea-msigdb.org/gsea/msigdb/index.jsp) using the MSigDB database v7.2 (>1.3-fold, |RC log2 | > 2, *P* < 0.05).

The aortic transcriptomes of humans from the NCBI Gene Expression Omnibus (GEO; http://www.ncbi.nlm.nih.gov/geo) database (GSE43292, GSE12644, and GSE83453) were computed for the analysis of gene expression and Pearson’s correlations with GraphPad Prism 7. The single-cell transcriptome of human patients (GSE159677) was utilized to extract each cell type in the aortas, including VSMCs, endothelial cells, macrophages, T-lymphocytes, B-lymphocytes, and NK cells, by marker genes. The genes of VSMCs were aligned and projected through uniform manifold approximation and projection (UMAP) to explore the scRNA-seq data. Visualization of gene expression was carried out in GraphPad Prism 7.

A luciferase reporter assay was performed as previously described^15^. Briefly, A7r5 cells were transfected with control or Cdon-overexpressing plasmids or infected with control- or shCdon-lentiviruses and β-catenin-responsive Top-Flash luciferase construct plasmid. Cells were analyzed for luciferase activity with a microplate luminometer according to the manufacturer’s protocol 24 h after transfection (Promega, Madison, USA).

### Alizarin red staining

Cells were washed with PBS and fixed with 4% PFA for 15 min at room temperature. The cells were then washed twice with deionized water and covered with 40 mM Alizarin Red S (Sigma-Aldrich, St. Louis, USA) at approximately pH 4.2 for 2 h at room temperature with gentle shaking. For removal of the unstained dye, the cells were washed three times with deionized water before images were obtained using a Nikon CELIPS TE-2000U (Nikon, Tokyo, Japan). For quantification of calcification, Alizarin Red S was extracted with 10% acetic acid for 30 min at room temperature, scraped into a microcentrifuge tube, vortexed, and incubated at 85 °C for 15 min. After chilling on ice for 5 min, the mixture was centrifuged at 20,000 × *g* for 15 min at 4 °C. The supernatant was transferred to a new tube, and 10% ammonium hydroxide was added to the supernatant. Absorbance was read in triplicate at 405 nm using a 96-well plate spectrophotometer^18^.

### Protein purification

HEK293T cells were transfected with recombinant proteins of Cdon fused with the Fc region of human IgG gamma listed as follows: Cdon-Fc; the entire ectodomain fused to Fc, Ig2-Fc; Fc fusion protein with Ig2 domain^15^. Seventy-two hours after transfection, the cells were pelleted by centrifugation at 2000 × *g* for 10 min. The supernatant was then filtered (0.45 μm) and subjected to Protein A antibody purification^22^. In brief, the supernatant was incubated with Protein A agarose (Millipore, Burlington, USA) for 1 h at room temperature under constant rotation. Bound proteins were eluted with 0.1 M citric acid (pH 3.0) and immediately neutralized with 1 M Tris-HCl (pH 8.0). Eluates were concentrated using Amicon Ultra4 centrifugal filters (MWCO 3 K, EMD Millipore, Burlington, USA) and dialyzed against PBS. Protein concentrations were determined using A280 measurements. Protein purity and integrity were assessed by sodium dodecyl sulfate-polyacrylamide gel electrophoresis.

### Statistical analysis

Values are the means ± SEMs or SDs, as noted. Statistical significance was calculated by paired or unpaired Student’s *t*-test or one-tailed analysis of variance (ANOVA) followed by Tukey’s test (GraphPad Prism software, v7). Differences were considered significant at *P* < 0.05.

## Results

### Cdon expression is reduced in calcified aortas and VSMCs during osteogenic conversion

**Fig. 1:**
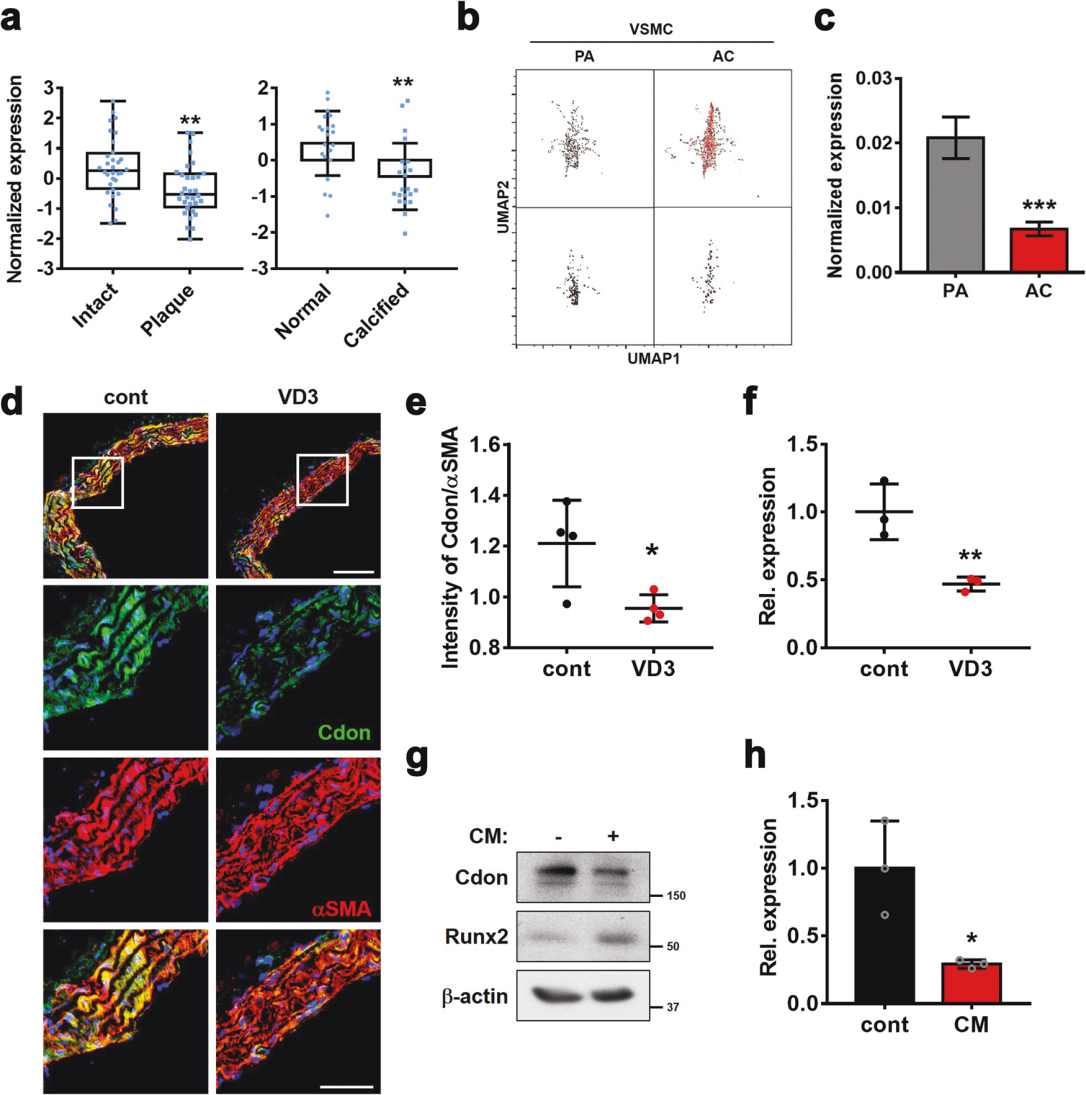
Cdon expression is reduced in calcified aortas. **a** Scatterplots of *Cdon* expression in aortic samples from patients with atherosclerotic plaques (GSE43292, *n* = 32) and calcified aortas (GSE12644 and GSE83453, *n* = 22). Statistical significance was determined with a two-tailed Student’s *t*-test. **P < 0.01. **b** Uniform manifold approximation and projection (UMAP) visualization of VSMCs (upper box: all genes in VSMCs, bottom box: only Cdon expression in VSMCs) in calcified atherosclerotic core plaques (AC) and patient-matched proximal adjacent portions (PA) of the carotid artery (GSE159677, *n* = 3). **c** *Cdon* expression in PA VSMCs and AC VSMCs (*n* = 3). Statistical significance was determined with a two-tailed Student’s *t*-test. ***P < 0.005. **d** Representative immunostaining images of Cdon and αSMA in aortas injected with VD3. Scale bar: 100 μm (top) and 50 μm (bottom). **e** Quantification of the intensity of Cdon fluorescence normalized to the intensity of αSMA, as shown in Panel **d** (*n* = 4). Data represent the means ± SEMs analyzed by Student’s *t*-test. *P < 0.05. **f** Relative RNA expression level of *Cdon* in aortic samples from VD3-injected mice (*n* = 3). Data represent the means ± SEMs analyzed by Student’s *t*-test. **P < 0.01. **g** Immunoblot analysis of VSMCs cultured with CM. **h** The relative RNA expression of *Cdon* in CM-treated VSMCs. Data represent the means ± SEMs analyzed by Student’s *t*-test. *P < 0.05.

The immunostaining data of mouse aortas revealed the expression of Cdon in the medial smooth muscle region, as well as in intimal endothelial cell regions (Supplementary Fig. 1a). Next, we analyzed the datasets (GSE43292, GSE12644, and GSE83453) obtained from aortas isolated from patients with atherosclerosis and stenosis for *Cdon* expression (Fig. 1a), together with aortic calcified markers such as *Runx2*, *ALPL* (alkaline phosphatase), and *CD68* (Supplementary Fig. 1b). Cdon was significantly downregulated in atherosclerotic plaques and calcified aortas, while the other coreceptors of Shh, such as *Boc* and *Gas1*, were not significantly altered. To further examine the expression pattern of Cdon in each cell type of aortas, we examined scRNA data (GSE159677) obtained from calcified atherosclerotic plaques (AC) and proximal adjacent portions (PA) of three patients (Fig. 1b). During the development of atherosclerosis, VSMCs, normally located in tunica media of arteries, migrate to the tunica intima and function as foam cells, resulting in the formation of atherosclerotic plaques^23,24^. Single-cell transcriptome analysis revealed a decrease in *Cdon* expression in VSMCs, endothelial cells, and macrophages (Fig. 1c and Supplementary Fig. 1c). Taken together, these data identify the potential role of Cdon in VSMCs during vascular pathogenesis.

We investigated the role of Cdon in vascular calcification by using a vascular calcification mouse model generated by subcutaneous administration of VD3 for 3 consecutive days (Supplementary Fig. 2a). Treatment with VD3 for 3 days induced aortic stiffness without overt cardiac dysfunction (Supplementary Fig. 2b). In the VD3-injected aortas, the osteogenic conversion was shown by the increase in osteogenic genes such as *Runx2* and the calcified area stained by Von Kossa. However, the expression of foam cell markers such as *CD146* and *CD68* was not significantly altered (Supplementary Fig. 2c, d). Consistent with the open data analysis, calcified aortas induced by VD3 had decreased Cdon transcript and protein levels relative to control aortas (Fig. 1d–f). To further assess the effect of calcification on Cdon expression, we treated A7r5 VSMCs with CM to mimic in vivo calcification (Supplementary Fig. 2e, f). Similar to the in vivo calcification model, CM treatment also reduced Cdon expression while elevating the expression of osteogenic markers such as *Runx2* and *ALPL* (Fig. 1g, h and Supplementary Fig. 2g). Collectively, these data suggest a potential role for Cdon in the prevention of vascular calcification.

### VSMC-Cdon ablation in mice exacerbates VD3-induced aortic calcification

**Fig. 2:**
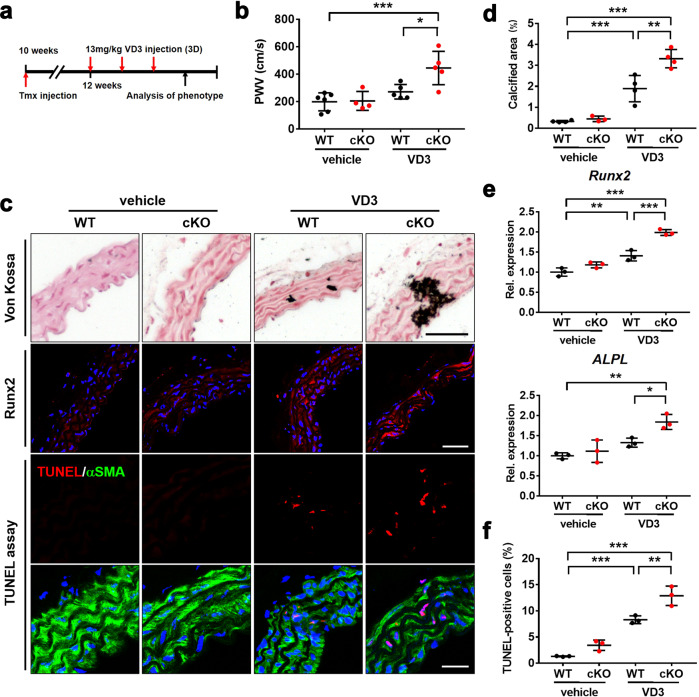
Cdon deficiency aggravates aortic stenosis and calcification. **a** The experimental scheme to induce vascular calcification in WT or Cdon-deficient aortas. cKO mice were generated by tmx injection. WT or cKO mice were injected subcutaneously with VD3 three times as indicated. **b** Echocardiographic parameters for aortic stenosis: pulse wave velocity (PWV) in the vehicle- or VD3-injected WT or cKO mice (*n* = 5). Data represent the means ± SEMs analyzed by one-way ANOVA. *P < 0.05, ***P < 0.005. **c** Representative images of Von Kossa staining and immunostaining for Runx2 and TUNEL assays in the vehicle- or VD3-treated WT or cKO aortas. Scale bar: 100 μm (top), 40 μm (middle), and 20 μm (bottom). **d** Quantification of the calcified area in the vehicle- or VD3-treated aortas as shown in panel (**c**) (*n* = 4). Data represent the means ± SEMs analyzed by one-way ANOVA. **P < 0.01, ***P < 0.005. **e** Relative RNA expression of osteogenic markers in the vehicle- or VD3-treated WT or cKO aortas (*n* = 3). Data represent the means ± SEMs analyzed by one-way ANOVA. *P < 0.05, **P < 0.01, ***P < 0.005. **f** Quantification of the number of TUNEL-positive VSMCs in the WT or cKO mice treated with vehicle or VD3 (*n* = 3), as shown in panel (**c**). Data represent the means ± SEMs analyzed by one-way ANOVA. **P < 0.01. ***P < 0.005.

Next, we examined the effect of Cdon deficiency on VD3-induced vascular calcification by using smooth muscle-specific Cdon ablated mice. We generated mice bearing tmx-inducible Cre recombinase under the control of the VSMC-specific *SM22a* promoter (*Cdon*^*f/f;SM22a-Cre-ERT2*^). Smooth muscle-specific deletion of *Cdon* (cKO) was induced by intraperitoneal injection of tmx five times at 2-day intervals (Supplementary Fig. 3a). Cdon protein levels were decreased in the cKO aortas, while other cKO tissues did not display any changes in Cdon levels compared to the WT tissues (Supplementary Fig. 3b, c). The residual Cdon proteins in the aorta might be from other cell types, such as endothelial cells. For induction of vascular calcification, VD3 was subcutaneously administered to mice for 3 days after 1 week of tmx injection (Fig. 2a). While Cdon deletion alone did not induce vascular calcification, it aggravated aortic stiffness in response to VD3 injection without cardiac dysfunction (Fig. 2b and Supplementary Fig. 4). The calcified area induced by VD3 was also increased in the cKO mice compared to the f/f mice (Fig. 2c, d). The expression of *Runx2* and *ALPL* was elevated by VD3 injection in both the control and cKO aortas, but it was elevated to a greater degree in the VD3-injected cKO aortas (Fig. 2e). Interestingly, Cdon deficiency did not affect the expression of *α-SMA*. Rather, VD3 administration led to a decline in the level of *α-SMA* in both the control and cKO aortas (Supplementary Fig. 5). The cKO aortas also displayed a greater number of cell deaths triggered by VD3 injection than the WT aortas, as evidenced by TUNEL-positive cells (Fig. 2c, f). Collectively, these data show that Cdon deletion in VSMCs exacerbates vascular calcification.

### Cdon depletion induces osteogenic transdifferentiation of VSMCs

**Fig. 3:**
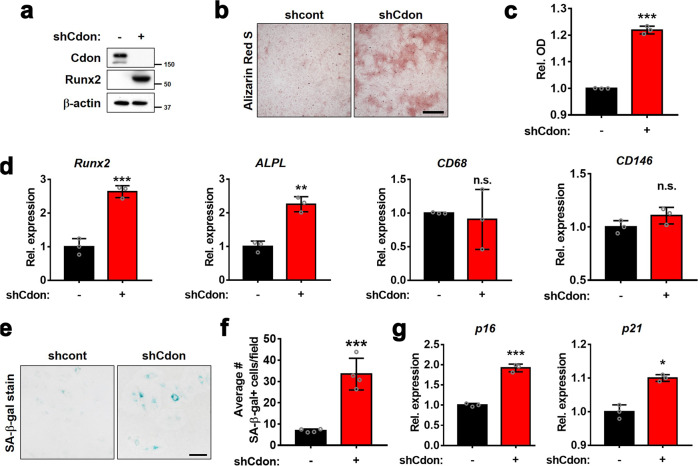
Cdon depletion induces the transition of VSMCs to osteoblast-like cells. **a** Immunoblot analysis of VSMCs infected with shCont- or shCdon-expressing lentivirus. **b** Representative Alizarin Red staining images of the shCont- or shCdon-transduced VSMCs. Scale bar:100 μm. **c** Quantification of Alizarin Red staining in the VSMCs transduced with shCont- or shCdon-lentiviruses shown in panel (**b**). (*n* = 3). Data represent the means ± SEMs analyzed by Student’s *t*-test. ***P < 0.005. **d** Relative transcript levels of osteogenic markers and foam cell markers in control or Cdon-depleted VSMCs (*n* = 3). Data represent the means ± SEMs analyzed by Student’s *t*-test. **P < 0.01, ***P < 0.005. n.s. = not significant. **e** Representative images of senescence-associated β-galactosidase (SA-β-gal) staining in the control or Cdon-deficient VSMCs. Scale bar: 100 μm. **f** Quantification of the number of SA-β-gal-positive cells shown in panel (**e**) (*n* = 4). Data represent the means ± SEMs analyzed by Student’s *t*-test. ***P < 0.005. **g** Relative transcript levels of cellular senescence markers in the control or Cdon-deficient VSMCs (*n* = 3). Data represent the means ± SEMs analyzed by Student’s *t*-test. *P < 0.05, ***P < 0.005.

To further verify the effect of Cdon depletion in smooth muscle cells, we transduced A7r5 cells with the control or Cdon shRNA-expressing lentiviruses and subjected them to calcification analysis. Interestingly, Cdon depletion alone elevated the Runx2 protein level and Alizarin Red staining intensity without calcification induction (Fig. 3a–c). Similarly, Cdon depletion induced an increase in the transcript levels of osteogenic markers such as *Runx2* and *ALPL*, while the gene expression related to foam cell markers (*CD68* and *CD146*) was not significantly altered relative to that of the control-infected cells (Fig. 3d). The osteogenic increase in the Cdon-depleted cells without calcification triggers might be due to the stresses caused by lentiviral infection. When we initially suppressed Cdon expression by using siCdon, which targets the same sequences as shCdon, the depletion did not induce calcification changes, as indicated by Runx2 expression under normal conditions (Supplementary Fig. 6). In addition, since control virus infection did not show any signs of calcification in A7r5 cells, Cdon depletion might sensitize VSMCs to other stresses to trigger calcification. These data indicate that Cdon plays a critical role in the maintenance of VSMC characteristics. Previous studies have shown that cellular senescence is a prominent driver of vascular dysfunction^25^. A recent study showed that Cdon ablation in skeletal muscle stem cells augments cellular senescence upon muscle injury, leading to impaired muscle regeneration^17^. Thus, we assessed whether Cdon depletion causes cellular senescence of VSMCs by using senescence-associated β-galactosidase (SA-β-gal) staining. The Cdon-depleted VSMCs had an approximately 3.5-fold increase in SA-β-gal-positive cells relative to the control cells (Fig. 3e, f). The expression of senescence markers such as *p16* and *p21* was also increased in the Cdon-depleted VSMCs (Fig. 3g). Taken together, these data suggest that Cdon depletion facilitates the osteogenic conversion and cellular senescence of VSMCs.

### Cdon deficiency in the aortas results in alterations of genes related to immune response, cell death, cell-to-cell adhesion, and Wnt signaling

**Fig. 4:**
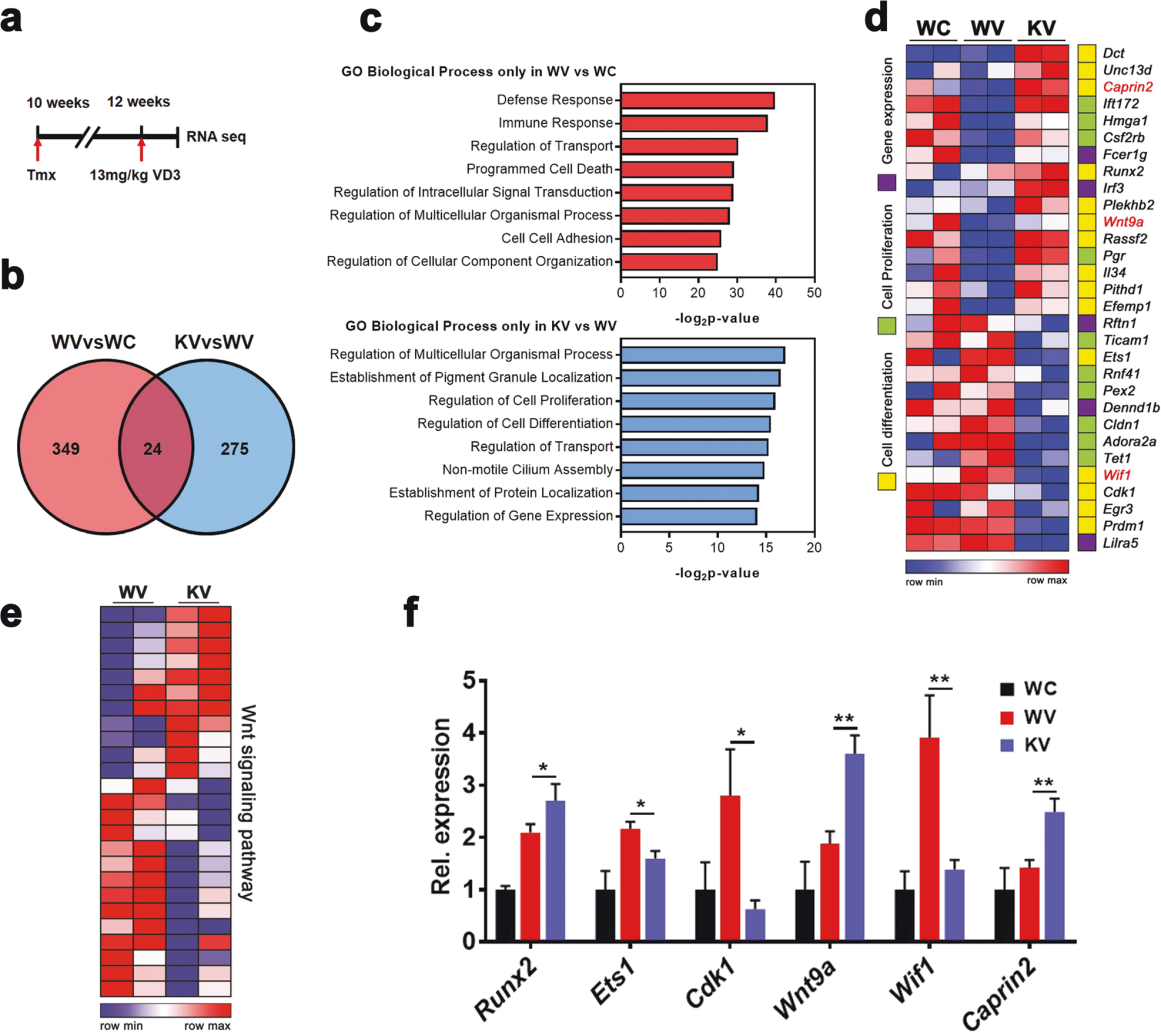
Cdon-deficient aortas display alterations in genes related to cell proliferation and differentiation accompanied by Wnt signaling components. **a** The experimental scheme for the RNA-sequencing analysis using RNAs from the aortas of the vehicle-treated WT (WC), VD3-treated WT (WV), and VD3-treated cKO (KV) groups for 1 day. **b** Venn diagram for shared or distinct gene numbers among the differentially expressed genes in WV vs. WC and KV vs. WV (*n* = 2, with biological repeats, >1.3-fold, normalized with |RC|log2 > 2, *p* < 0.05). **c** Enriched GO terms in biological process terms of 349 VD3-mediated unique genes (top) or 275 Cdon-dependent VD3-regulated genes (bottom) by GSEA. **d** Heatmap showing the gene expression pattern of three gene sets (cell differentiation, cell proliferation, and gene expression). The red letter indicates the regulator of the Wnt signaling pathway. **e** Comparison of gene expression levels of the Wnt signaling pathway between WV and KV. **f** Relative transcript levels (*n* = 3). Data represent the means ± SEMs analyzed by one-way ANOVA. *P < 0.05, ***P < 0.01.

To analyze Cdon-mediated molecular mechanisms in vascular calcification, we performed RNA sequencing with the WT and cKO aortas one day after VD3 injection (Fig. 4a). There were 373 differentially expressed genes (DEGs) in the VD3-treated WT (WV) vs. vehicle-treated WT (WC) groups and 299 DEGs in the VD3-treated cKO (KV) vs. WV groups (>1.3-fold, average of normalized RC log2 > 2, *P* value < 0.05 with biological repeats, Fig. 4b). There were 24 DEGs that overlapped between WV vs. WC and KV vs. WV, and we hypothesized that those genes are not Cdon-related genes. Thus, each DEG in WV vs. WC or KV vs. WV, excluding the overlapping genes, was further analyzed by using gene set enrichment analysis (GSEA) (Fig. 4c). The data revealed that genes related to the immune response, cell death, and cell-to-cell adhesion were significantly altered only in WV vs. WC, while genes implicated in cell proliferation, cell differentiation, and gene expression regulation were altered in KV vs. WV (Fig. 4c, d). Notably, in KV vs. WV, genes related to Wnt signaling regulation, such as cytoplasmic activation/proliferation-associated protein 2 (*Caprin2*), Wnt inhibitory factor 1 (*Wif1*), and *Wnt9a*, were altered. Consistently, the Cdon-deficient aortas exhibited altered Wnt activities in response to VD3 (Fig. 4e), which was further confirmed by quantitative RT-PCR analysis (Fig. 4f). To further examine the relationship between Cdon and Wnt signaling, we assessed the aortic transcriptome from patients suffering from atherosclerosis and aortic stenosis (GSE43292, GSE12644, and GSE83453) (Supplementary Fig. 7a). We found a negative correlation between Cdon and Wnt target genes, including *Wnt3*, *Ctnnb1*, *Fzd1*, and *Axin2* (Supplementary Fig. 7b). Taken together, these data suggest that Cdon deficiency causes changes in VSMC proliferation and differentiation via Wnt signaling.

### Cdon depletion enhances Wnt signaling, leading to VSMC calcification

**Fig. 5:**
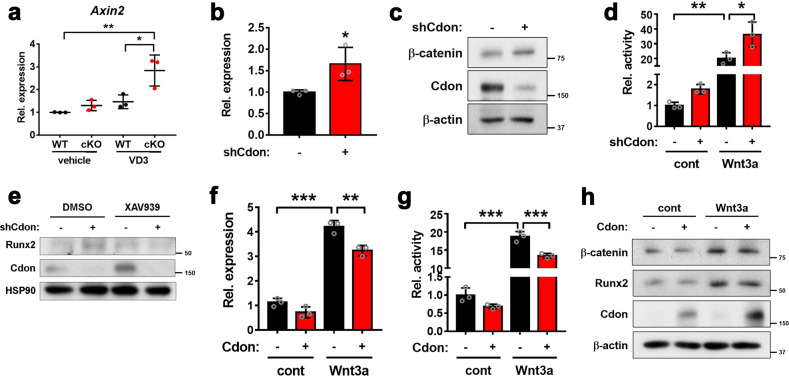
Cdon-depleted VSMCs activate the Wnt signaling pathway. **a** Relative *Axin2* transcript levels in the vehicle- and VD3-treated aortas (*n* = 3). Data represent the means ± SEMs analyzed by one-way ANOVA. *P < 0.05, **P < 0.01. **b** Relative *Axin2* transcript levels in the control or Cdon-deficient cells (*n* = 3). Data represent the means ± SEMs analyzed by Student’s *t*-test. *P < 0.05. **c** Immunoblot analysis of the shCont- or shCdon lentivirus-infected VSMCs. **d** Top-flash reporter activity of the control or Cdon-depleted VSMCs in response to Wnt3a (*n* = 3). Data represent the means ± SEMs analyzed by one-way ANOVA. *P < 0.05, **P < 0.01. **e** Immunoblot analysis of the shCont- or shCdon-infected VSMCs in response to DMSO or XAV939. **f** Relative *Axin2* expression in control- or Cdon-overexpressing VSMCs treated with Wnt3a (*n* = 3). Data represent the means ± SEMs analyzed by one-way ANOVA. **P < 0.01, ***P < 0.005. **g** The TopFlash reporter activity of the control- or Cdon-overexpressing VSMCs in response to Wnt3a (*n* = 3). Data represent the means ± SEMs analyzed by Student’s *t*-test and one-way ANOVA. ***P < 0.005. **h** Immunoblot analysis of the control- or Cdon-overexpressing VSMCs in response to Wnt3a.

In an earlier study, we demonstrated that Cdon promotes neuronal differentiation by negatively regulating the Wnt signaling pathway^15^. Inspired by previous findings and the current results of RNA sequencing of Cdon-deficient aortas, we examined the interaction of Cdon with the Wnt signaling pathway in vascular calcification. First, we assessed the expression of the Wnt target gene *Axin2* in Cdon-deleted aortas. The *Axin2* transcript level was significantly elevated in both the WT and cKO aortas treated with VD3 but was significantly higher in the cKO aortas (Fig. 5a). Consistently, Cdon depletion in VSMCs caused a significant increase in *Axin2* expression compared to the control (Fig. 5b). The level of β-catenin was also substantially elevated in the Cdon-depleted VSMCs (Fig. 5c). Furthermore, the activity of the Topflash Wnt reporter was elevated in the Cdon-depleted VSMCs compared to the controls, and the difference between the Cdon-depleted VSMCs and the controls became more prominent in response to Wnt3a (Fig. 5d). To further clarify the effect of Cdon depletion on Wnt signaling-mediated calcification in VSMCs, we analyzed Runx2 expression in the shCont- or shCdon-infected cells after treatment with DMSO or XAV939 (Fig. 5e). The results showed that the increase in Runx2 under Cdon-depleted conditions was diminished when Wnt signaling was inhibited by XAV939, even under Cdon knockdown conditions. In contrast, Cdon overexpression reduced *Axin2* expression and Wnt reporter activities under both basal and Wnt3a-treated conditions (Fig. 5f, g). Furthermore, Cdon overexpression attenuated the increase in β-catenin and Runx2 proteins in response to Wnt3a compared to that of the control cells (Fig. 5h). Taken together, these data suggest that Cdon modulates Wnt signaling activation in VSMCs.

### Cdon overexpression attenuates the CM-induced osteogenic conversion of VSMCs

**Fig. 6:**
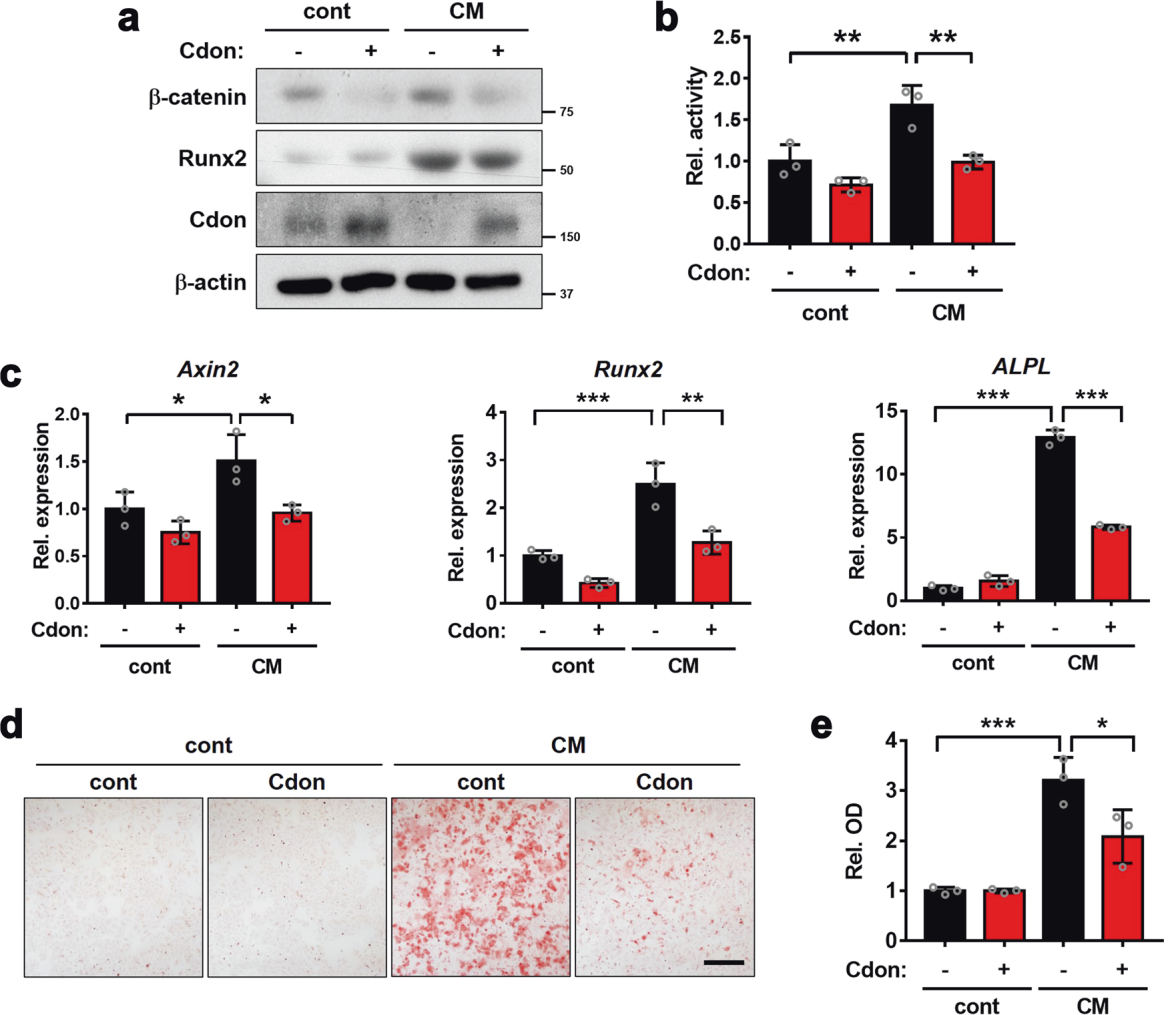
Cdon overexpression attenuates the osteogenic conversion of VSMCs in response to CM. **a** Immunoblot analysis of the control- or Cdon-overexpressing VSMCs in response to CM. **b** TopFlash reporter activity in control- or Cdon-overexpressing VSMCs in response to CM (*n* = 3). Data represent the means ± SEMs analyzed by one-way ANOVA. **P < 0.01. **c** Relative RNA expression of *Axin2*, *Runx2*, and *ALPL* in control- or Cdon-overexpressing VSMCs treated with CM (*n* = 3). Data represent the means ± SEMs analyzed by one-way ANOVA. *P < 0.05, **P < 0.01, and ***P < 0.005. **d** Alizarin Red staining images of the control- or Cdon-overexpressing VSMCs in response to CM. Scale bar: 100 μm. **e** Quantification of Alizarin Red staining in the VSMCs shown in panel (**d**) (*n* = 3). Data represent the means ± SEMs analyzed by one-way ANOVA. *P < 0.05, ***P < 0.005.

Since Cdon expression is decreased in calcified VSMCs, we examined whether Cdon overexpression attenuates CM-induced osteogenic conversion. Cdon-overexpressing VSMCs exhibited reduced levels of Runx2 and β-catenin proteins in response to CM (Fig. 6a). In addition, CM treatment significantly elevated Wnt-reporter activity, which was attenuated by Cdon overexpression (Fig. 6b). Cdon overexpression also blunted the expression of *Axin2*, *Runx2*, and *ALPL* in response to CM (Fig. 6c). Consistently, Cdon overexpression inhibited CM-induced osteogenic transdifferentiation of VSMCs (Fig. 6d, e). Taken together, these data suggest that Cdon overexpression suppresses the Wnt signaling pathway and VSMC calcification.

### Cdon attenuates osteogenic conversion by inhibiting the Wnt signaling pathway

**Fig. 7:**
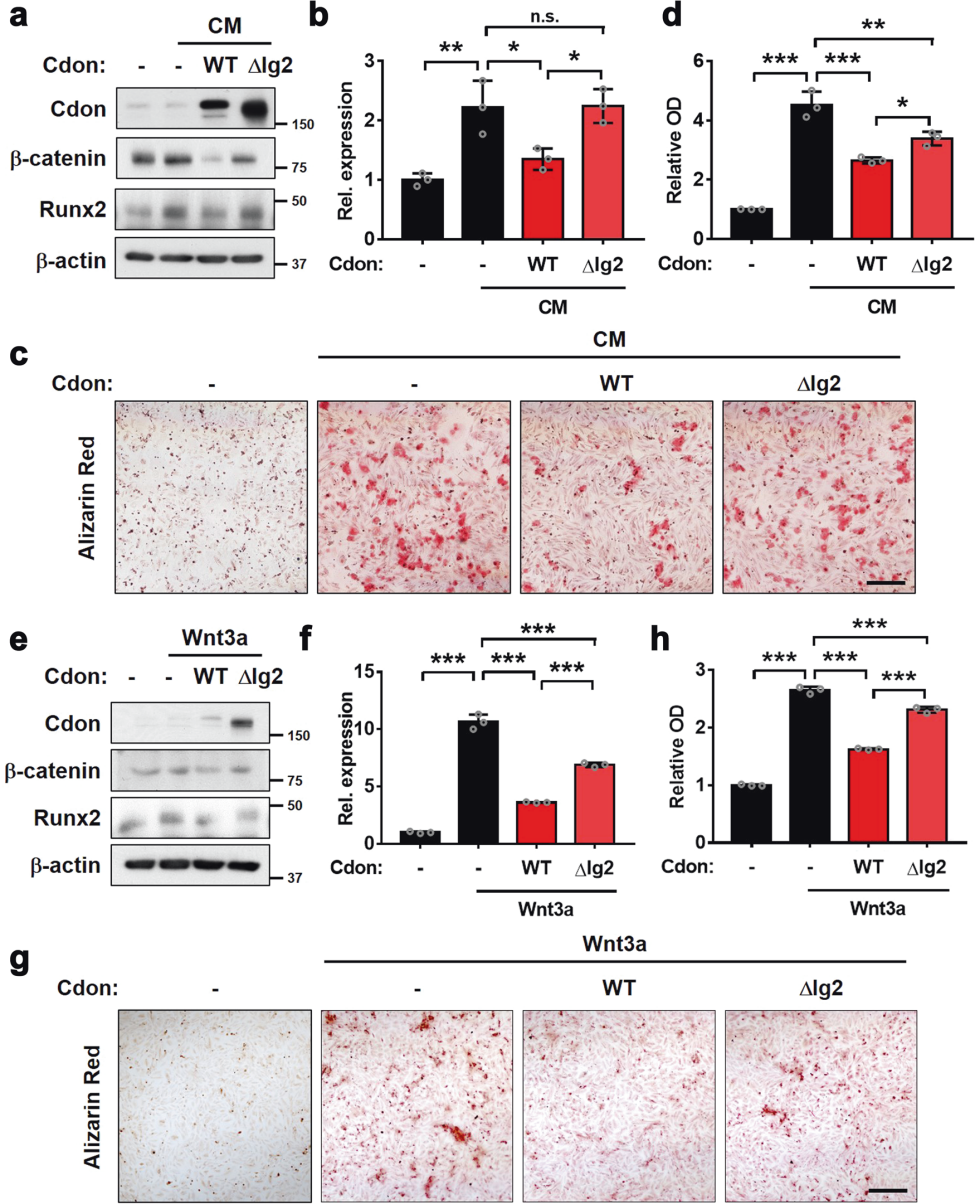
The deletion of the Ig2 domain of Cdon fails to block VSMC osteogenic conversion. **a** Immunoblot analysis of the VSMCs expressing control, full-length Cdon or Ig2 domain-deleted Cdon (ΔIg2) treated with vehicle or CM. **b** Relative *Axin2* expression in control-, Cdon-, or ΔIg2-expressing VSMCs in response to CM (*n* = 3). Data represent the means ± SEMs analyzed by one-way ANOVA. n.s. = not significant, *P < 0.05, **P < 0.01. **c** Alizarin Red staining of the control-, Cdon-, or ΔIg2-expressing VSMCs in response to CM. Scale bar: 100 μm. **d** Quantification of Alizarin Red staining shown in panel (**c**) (*n* = 3). Data represent the means ± SEMs analyzed by one-way ANOVA. *P < 0.05, ***P < 0.005, ***P < 0.005. **e** Immunoblot analysis of the VSMCs expressing control, full-length Cdon or Ig2 domain-deleted Cdon (ΔIg2) treated with vehicle or Wnt3a. **f** Relative *Axin2* expression in the control-, Cdon-, or ΔIg2-expressing VSMCs in response to Wnt3a (*n* = 3). Data represent the means ± SEMs analyzed by one-way ANOVA. ***P < 0.005. **g** Alizarin Red staining of the control-, Cdon-, or ΔIg2-expressing VSMCs in response to Wnt3a. Scale bar: 100 μm. **h** Quantification of Alizarin Red staining shown in panel (**g**) (*n* = 3). Data represent the means ± SEMs analyzed by one-way ANOVA. ***P < 0.005.

We have previously reported that Cdon suppresses the Wnt signaling pathway through interaction with the Wnt coreceptor LRP6 mediated by its second immunoglobulin domain (Ig2)^15^. Thus, we examined the effect of wild-type Cdon (WT) and a deletion mutant for the second Ig domain (ΔIg2) on CM-mediated osteogenic conversion. In contrast to the suppressive effect of WT on β-catenin levels in response to CM, the ΔIg2 mutant failed to fully repress the induction of β-catenin and Runx2 levels (Fig. 7a). In addition, the ΔIg2 mutant failed to blunt *Axin2* expression, which was greatly elevated by CM but attenuated by WT expression (Fig. 7b). Furthermore, the inhibitory effect of ΔIg2 mutant expression on the CM-induced osteogenic conversion of VSMCs was smaller than that of WT expression (Fig. 7c, d). Consistent with previous results, WT overexpression attenuated the increase in β-catenin and Runx2 by Wnt3a treatment, while ΔIg2 expression failed to suppress this increase (Fig. 7e). Similarly, the *Axin2* increase induced by Wnt3a was abrogated by WT, while the ΔIg2 mutant failed to fully suppress this increase (Fig. 7f). Consistently, WT overexpression reduced Wnt3a-induced osteogenic conversion, while ΔIg2 expression failed to do so (Fig. 7g, h). Collectively, these data indicate that Cdon has a preventive role against VSMC calcification through Wnt signaling inhibition.

### Cdon-Ig2-Fc fusion protein exhibits a preventive effect on VSMC calcification

**Fig. 8:**
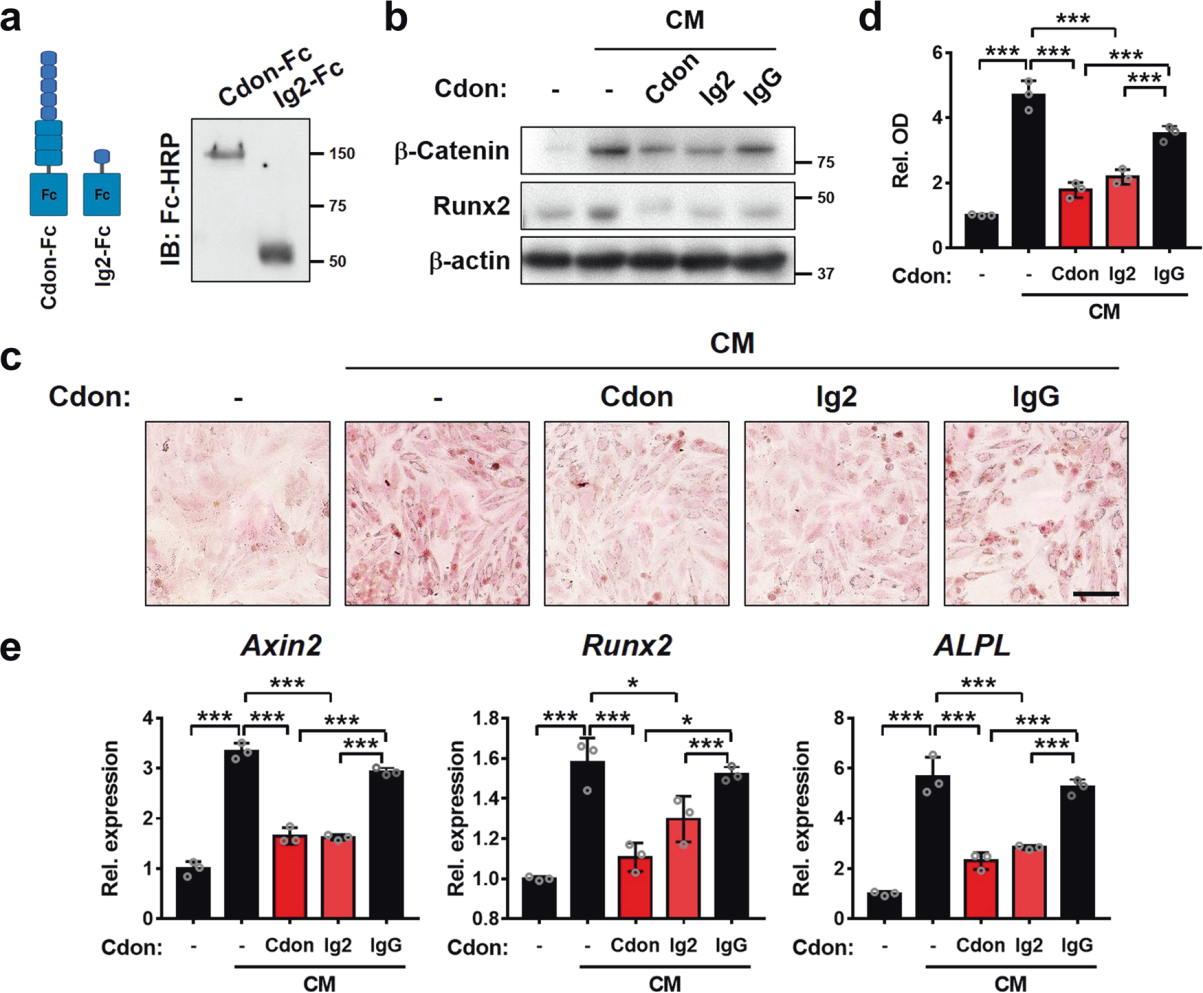
The Ig2 domain of Cdon is sufficient to attenuate the osteogenic conversion of VSMCs. **a** Immunoblot analysis of Cdon-Fc (the entire Cdon ectodomain fused to Fc) and Ig2-Fc (Ig2 domain fused to Fc) purified from cell supernatants. **b** Immunoblot analysis of VSMCs treated with CM and each purified protein. IgG served as a control. **c** Alizarin Red staining images of the VSMCs treated with control or CM in combination with Cdon-Fc, Ig2-Fc, or IgG. Scale bar = 100 μm. **d** Quantification of Alizarin Red staining shown in panel (**c**) (*n* = 3). Data represent the means ± SEMs analyzed by one-way ANOVA. ***P < 0.005. **e** Relative transcript levels of *Axin2*, *Runx2*, and *ALPL* in the VSMCs treated with control or CM in combination with Cdon-Fc, Ig2-Fc, or IgG.

Next, we further examined the effect of the secreted recombinant proteins of the Cdon ectodomain (Cdon-Fc) or Ig2 domain (Ig2-Fc) fused to the Fc domain of human IgG gamma. The expression of recombinant proteins was validated in the supernatants of the cells transfected with Cdon-Fc or Ig2-Fc by immunoblot analysis (Fig. 8a). The treatment of VSMCs with purified Cdon-Fc or Ig2-Fc (10 μg/ml) also blocked CM-induced β-catenin activation and Runx2 induction compared to human IgG as the negative control (Fig. 8b). CM-induced osteogenic conversion was also attenuated by Cdon-Fc or Ig2-Fc but not by control human IgG (Fig. 8c, d). Furthermore, Cdon-Fc or Ig2-Fc treatment attenuated CM-induced *Axin2*, *Runx2*, and *ALPL* expression, while control IgG did not show any effect (Fig. 8e). Taken together, these data suggest that the Ig2 domain of Cdon is sufficient to suppress Wnt signaling and VSMC calcification. In summary, Cdon plays a critical role in Wnt signaling suppression, thereby preventing vascular calcification.

## Discussion

Our data demonstrate the critical role of Cdon in vascular calcification. Transcriptome data from aortas isolated from patients with atherosclerosis and stenosis revealed a significant decrease in Cdon levels in VSMCs. In agreement, Cdon was reduced in the aortas of mouse models of VD3-induced vascular calcification or in CM-treated VSMCs. Furthermore, VSMC-specific depletion of Cdon exacerbated VD3-induced vascular calcification, although the effect of Cdon needs to be confirmed by performing an in vivo study with Cdon transgenic mice. Likewise, Cdon depletion or overexpression in VSMCs increased or attenuated the Wnt signaling pathway, respectively. Therefore, Cdon is an important regulator of vascular remodeling.

Shh signaling induces the proliferation of VSMCs, an essential step for intimal hyperplasia and the pathogenesis of vascular diseases^26^. Since Cdon activates Shh signaling as a coreceptor^27^, we initially predicted that Cdon depletion might reduce the proliferation of VSMCs and vascular remodeling via reduced Shh signaling. Transcriptome analysis revealed alterations in genes related to cell proliferation and differentiation in Cdon-deficient aortas treated with VD3. However, the involvement of Shh signaling in vascular calcification of Cdon-deficient aortas is unclear. Genes related to Shh signaling were not greatly altered in Cdon-deficient aortas in response to VD3. Unlike the reduced expression of Cdon, the expression of other Shh signaling components, Boc, Gas1, and Shh, was also not significantly changed in the transcriptome data from aortas isolated from patients with atherosclerosis and stenosis. Furthermore, Cdon depletion increased cellular senescence and death in the medial region of the aorta in response to VD3-mediated calcification. These data support an independent role of Cdon in Shh signaling in vascular diseases.

BMP signaling plays a critical role in the osteogenic differentiation of VSMCs^28,29^. BMP2, one of the transforming growth factor-β family members, activates Runx2 in diverse cell types, including VSMCs, and plays a crucial role in bone repair^30,31^. Aortic transcriptome analysis revealed a negative correlation between Cdon and the BMP signaling pathway. However, there was no significant difference in BMP signaling in Cdon-overexpressing or Cdon-depleted VSMCs by reporter assays (data not shown). Thus, BMP signaling may not be directly involved in Cdon-mediated VSMC regulation.

In addition to BMP signaling, an inverse correlation between Cdon and Wnt target genes was observed in the aortic transcriptome of patients with atherosclerosis and stenosis. Wnt signaling is another major mechanism implicated in the osteogenic conversion of VSMCs. Consistent with the negative effects of Cdon on Wnt signaling in forebrain development^15^, Cdon deficiency in VSMCs resulted in Wnt signaling dysregulation and enhanced osteogenic conversion. Our previous study identified the second Ig region of the ectodomain of Cdon to be responsible for Wnt signaling suppression by interacting with LRP6^15^. Accordingly, the deletion of Ig2 in Cdon failed to block osteogenic conversion in response to CM or Wnt3a treatment, suggesting a requirement for Wnt inhibition by the Ig2 domain to prevent VSMC conversion. Consistently, the Fc fusion protein of the Ig2 domain exhibited a suppressive effect on Wnt signaling activity and a protective effect on vascular calcification. Thus, Cdon represents a potential therapeutic tool for vascular calcification via suppression of Wnt signaling activity. Since the Wnt signaling pathway is involved in diverse diseases, including cancers and neurodegenerative diseases, the regulatory mechanisms of Wnt signaling have been continuously investigated for the development of therapeutic strategies^32–34^. DKK (Dickkopf) proteins, Wnt regulatory molecules, bind to LRP5/6 coreceptors, thereby acting as functional antagonists of Wnt signaling^35^. Interestingly, inhibition of Wnt signaling by DKK plays a complex role in cardiovascular diseases. DKK1 is elevated in the plasma and lesions of patients with atherosclerosis and type 2 diabetes with cardiovascular diseases^36,37^. Excessive DKK1 can promote endothelial cell dysfunction associated with increased inflammation, likely contributing to atherosclerosis^38^. However, DKK1 can attenuate calcium deposition and the expression of RUNX2 in calcified aortas from chronic kidney disease in a similar manner to Cdon^39^. The opposing effects of DKK1 may be attributed to its distinct role dependent on the cellular context and Wnt-independent signaling activities. A recent study on Cdon in the endothelium reported its role as a negative regulator of desert hedgehog-driven endothelial integrity in inflammatory conditions^14^. Therefore, therapeutic strategies using Wnt signaling regulators will require high specificity to avoid undesirable outcomes. In the case of Cdon, its interaction with Hedgehog proteins is mediated by the third fibronectin domain^40^. Since the Ig2 domain of Cdon has been shown to be sufficient to distinctively target and suppress Wnt signaling activities, the use of the Ig2 domain of Cdon may serve as a specific suppressor of the Wnt signaling pathway while avoiding the intervention of other signaling pathways.

Accumulating evidence suggests that cellular senescence is closely linked with vascular pathogenesis^25,41^. Cellular senescence of VSMCs has been associated with the induction of osteogenic markers, including Runx2 and ALPL^42^. Thus, cellular senescence is considered a major risk factor for vascular calcification. Cdon-deficient VSMCs exhibit increased cellular senescence in response to calcification stress. Similarly, skeletal muscle stem cells deficient for Cdon display cellular senescence, which contributes to impaired regeneration^17^. The mechanism by which Cdon depletion causes cellular senescence is currently unclear. Decreased Wnt signaling activity has been generally linked with cellular senescence^43^, but a recent study reported that the canonical Wnt signaling pathway in chondrocytes induces cellular senescence associated with inflammation^44^. Further study is required to elucidate the exact role of Wnt signaling and Cdon in VSMC senescence. Here, we report the suppressive effects of the Ig2 domain of Cdon on the Wnt signaling pathway by interacting with LRP6, indicating that the Ig2 domain of Cdon could be a good candidate for therapeutic strategies in vascular diseases.

## Supplementary information

Supplementary material
