# Supplementary material for: Cdon suppresses vascular smooth muscle calcification via repression of the Wnt/Runx2 Axis
Source: Exp Mol Med. 2023 Jan 6;55(1):120–31. doi: 10.1038/s12276-022-00909-7 (PMC9898282; doi:10.1038/s12276-022-00909-7)
Supplement: Supplementary file 1 — Supplementary material [file 12276_2022_909_MOESM1_ESM.pdf]

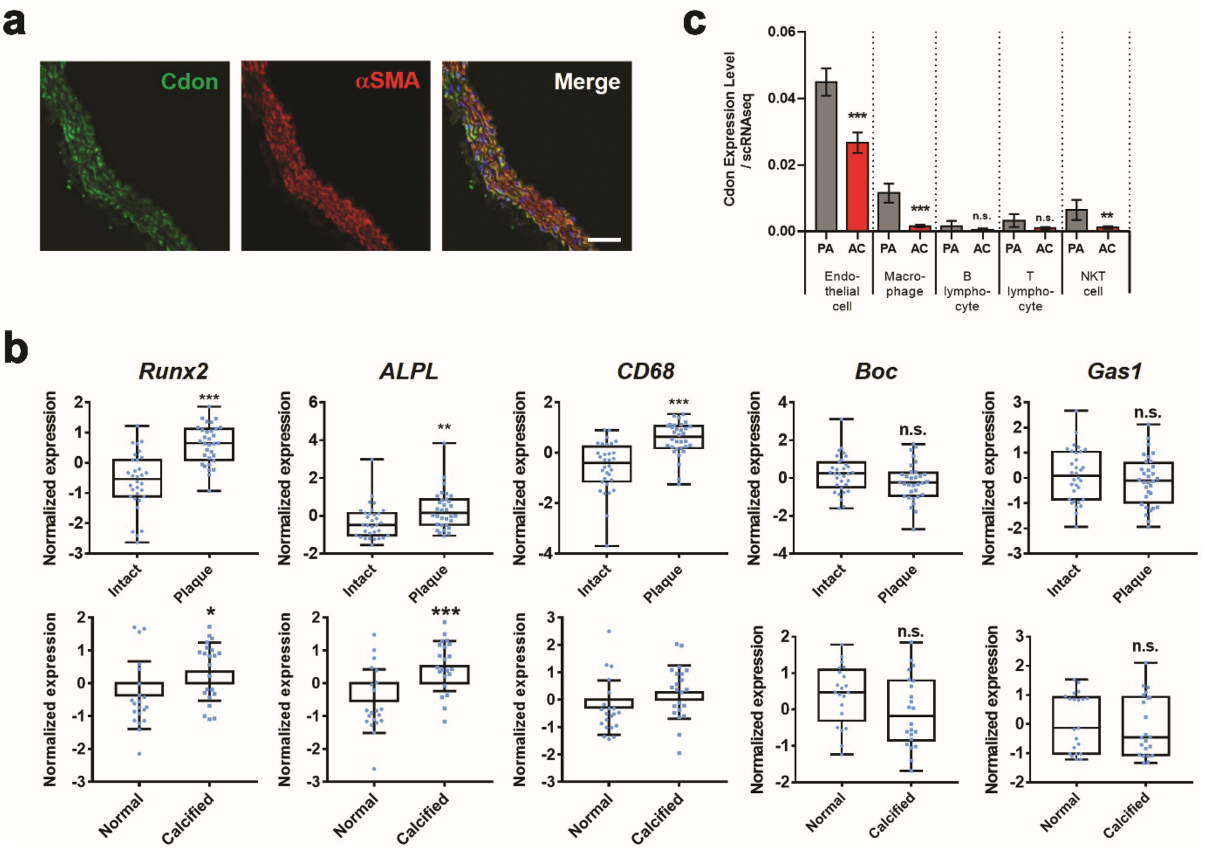

Supplementary Fig 1. The expression pattern of osteogenic markers, a foam cell marker and Sonic Hedgehog signaling coreceptors in atherosclerotic and calcified aortas.

(**a**) Representative immunostaining images of Cdon and αSMA in mouse aorta. Scale bar: 100µm. (**b**) Scatterplots showing the expression of *Runx2*, *ALPL*, *CD68*, *Boc* and *Gas1* in aortic samples from patients with atherosclerotic plaques (GSE43292, n=32) and calcified aortas (GSE12644 and GSE83453, n=22). Statistical significance is determined with two-tailed Student's t-test. n.s.=not significant. (**c**) *Cdon* expression in endothelial cells, macrophages, B-lymphocytes, T-lymphocytes, and NKT cells of calcified atherosclerotic core plaques (AC) and patient-matched proximal adjacent portions (PA) of carotid artery (GSE159677, n=3). Statistical significance is determined with two-tailed Student's t-test. *P<0.05, **P<0.01, ***P<0.005, n.s.=not significant.

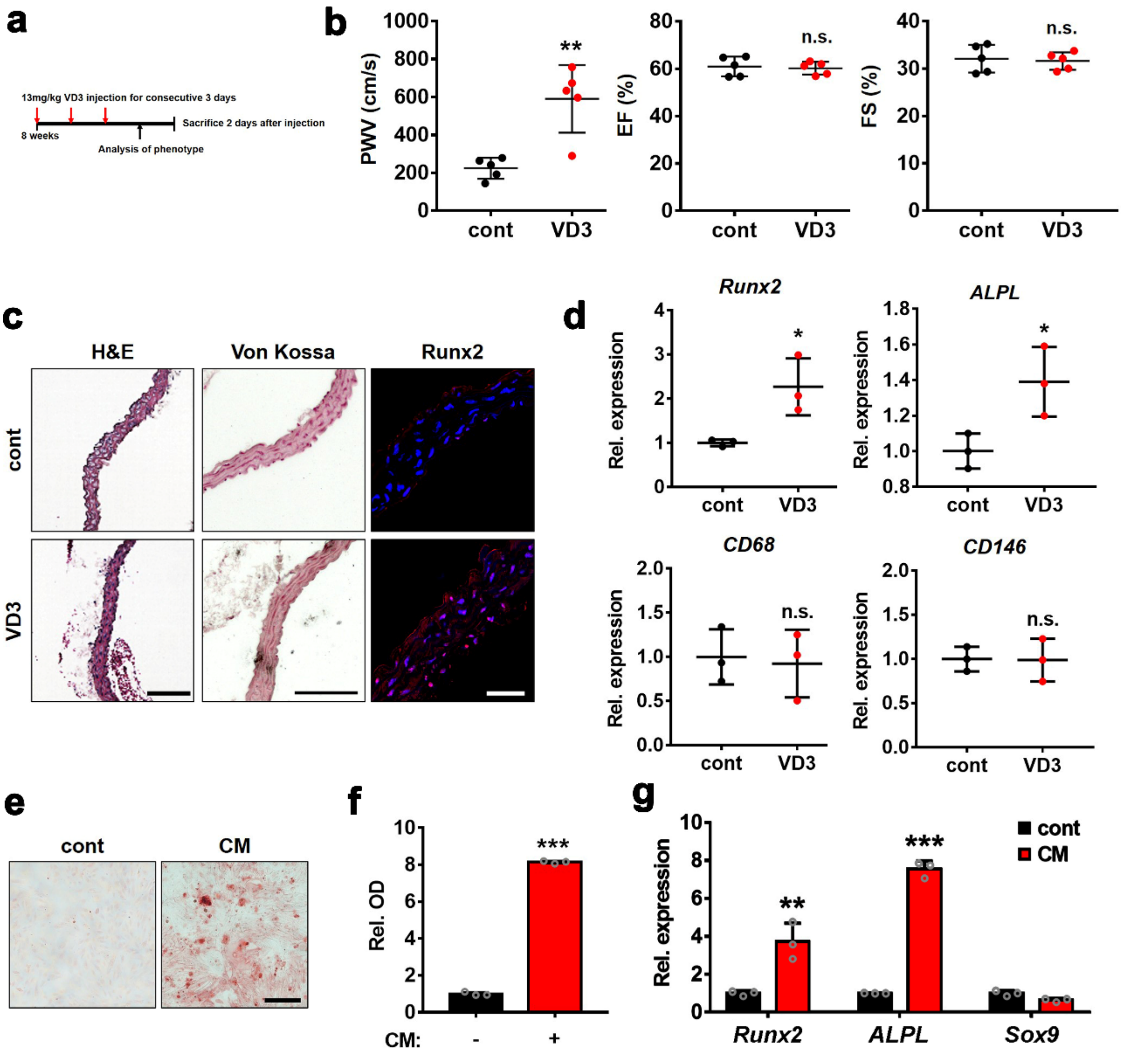

Supplementary Fig 2. Analysis of *in vivo* and *in vitro* vascular calcification models.

(**a**) The experimental scheme for inducing vascular calcification in mice. 8-week-old mice were administrated subcutaneously with VD3 for consecutive 3 days. (**b**) Echocardiographic parameters of VD3 injected mice for analyzing aortic stiffness and cardiac function (n=5): the pulse wave velocity (PWV), the ejection fraction (EF), and the fractional shortening (FS). Data represent means ±SEM analyzed by Student's t-test. n.s.=not significant, **$P<0.01$. (**c**) Representative images for histological staining and immunostaining. Scale bar: 100μm (Left), 200μm (Middle) and 50μm (Right). (**d**) Relative transcript levels of osteogenic and foam cell markers in aortas treated with VD3 (n=3). Data represent means ±SEM analyzed by Student's t-test. *$P<0.05$. (**e**) Alizarin red staining images of VSMCs treated with control or CM. Scale bar: 100μm. (**f**) Quantification of Alizarin Red staining shown in panel e. (n=3). Data represent means ±SEM analyzed by Student's t-test. ***$P<0.005$. (**g**) Relative transcript levels of osteogenic markers in VSMCs treated with CM. (n=3). Data represent means ±SEM analyzed by Student's t-test. **$P<0.01$, ***$P<0.005$.

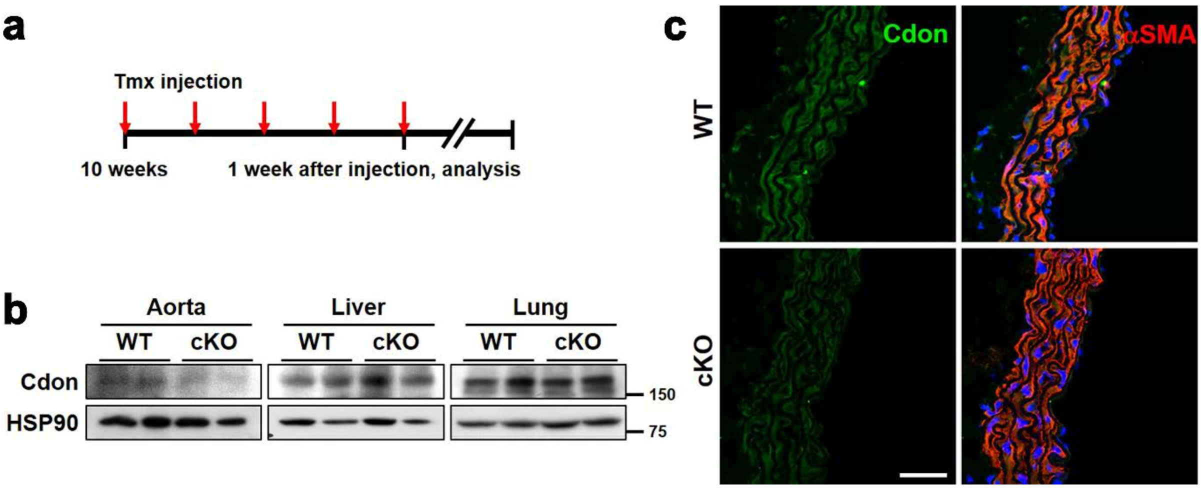

Supplementary Fig 3. Generation of mice ablated Cdon in VSMCs.

(**a**) To ablate Cdon in smooth muscles, *Cdon*$^{f/f;Sm22\alpha\text{-}CreERT2}$ mice (cKO) were administrated intraperitoneally with tmx as indicated. (**b**) Immunoblot analysis for Cdon expression in aorta, liver and lung from WT and cKO mice. (**c**) Immunostaining for Cdon and αSMA in aortas. Scale bar: 40μm.

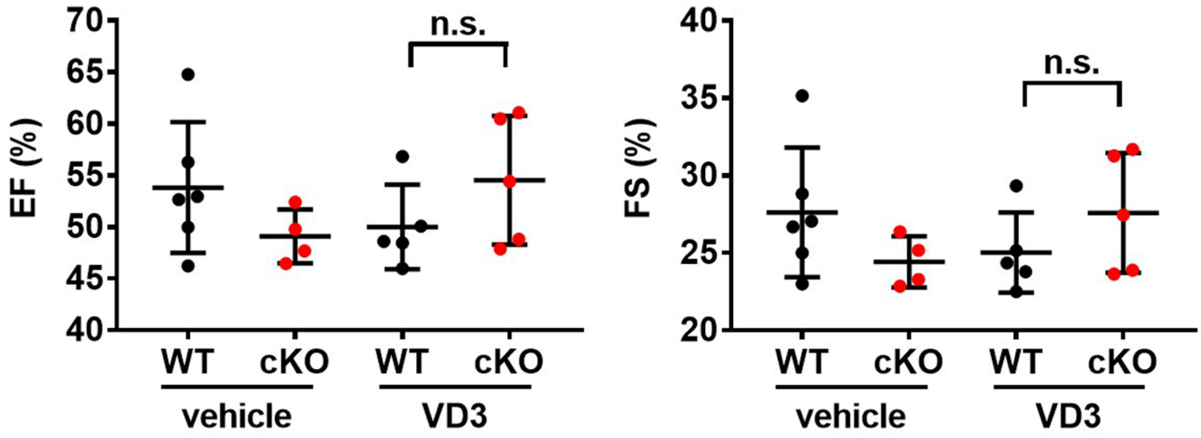

Supplementary Fig 4. Cardiac function is unaffected in cKO mice.

Echocardiographic parameters in WT and cKO mice treated with vehicle or VD3: the ejection fraction (EF) and the fractional shortening (FS) (n=5). Data represent means ±SEM analyzed by one-way ANOVA test. n.s.=not significant.

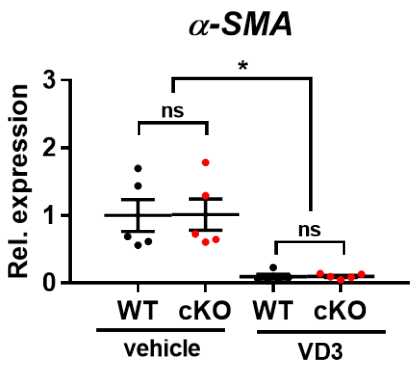

Supplementary Fig 5. Cdon deficiency does not influence on *α-SMA* expression.

Relative RNA expression of *α-SMA* in vehicle- or VD3-treated WT or cKO aortas (n=3). Data represent means ±SEM analyzed by one-way ANOVA test. *P<0.05, n.s.=not significant.

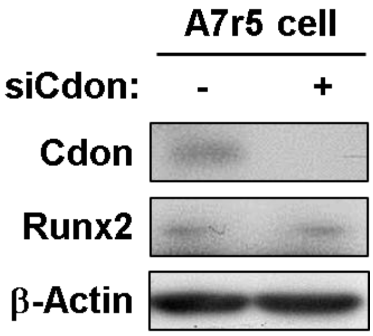

Supplementary Fig 6. Cdon deficiency without viral infection-induced stress does not induce calcification under the normal condition.

A7r5 VSMCs were transfected with siCdon, which targets the same sequence as shCdon and cultured under the normal cell culture condition. Western blot was performed with anti-Cdon or anti-Runx2 antibody. β-Actin was selected as a loading control.

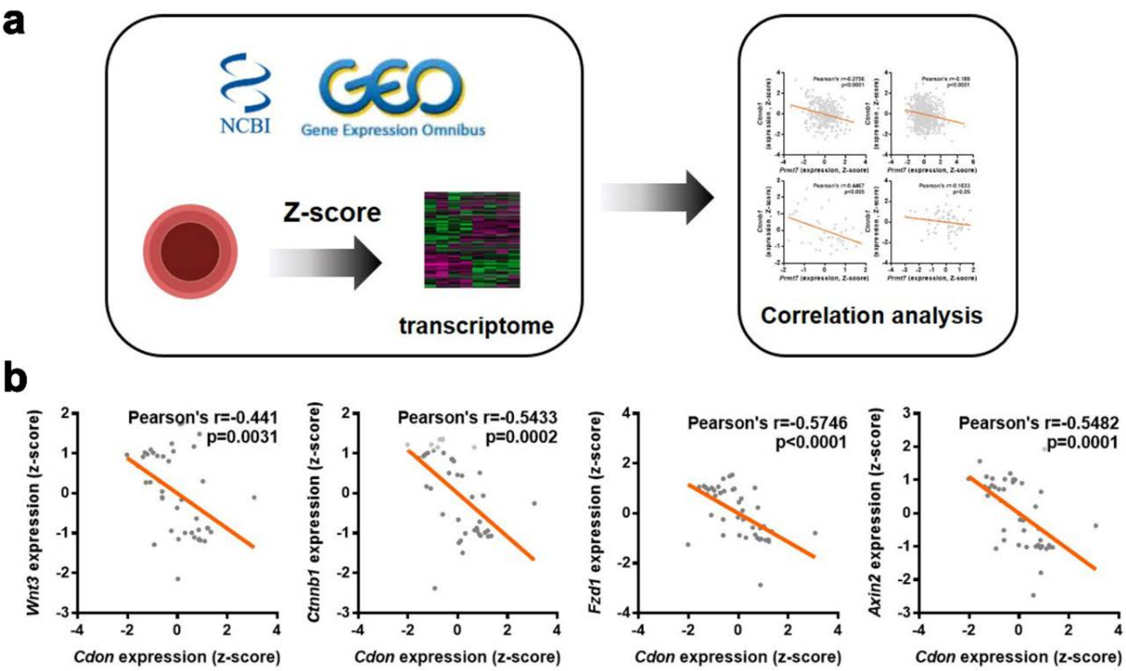

Supplementary Fig 7. The negative correlation between Cdon and Wnt signaling genes in aorta.

(**a**) The scheme of stepwise workflow of an aortic transcriptome analysis: collect the gene expression profile for aortas, normalize the value of gene expression to Z-score, and analyze the correlation between *Cdon* and potential target genes. (**b**) The scatter plots presenting the correlated expression patterns between *Cdon* (X-axis) and Wnt signaling genes like *Wnt3*, *Ctnnb1*, *Fzd1*, and *Axin2* (Y-axis).

Supplementary Table 1. The primary antibodies used in this study

Antigen	Host	Cat. No.	Manufacturer
**α-SMA**	R	Ab5694	Abcam
**β-Actin**	R	#4970	Cell signaling technology
**β-Catenin**	M	Sc7963	Santa Cruz
**Cdon**	G	AF2429	R&D systems
**HSP90**	R	Sc-7947	Snta Cruz
**Runx2**	M	D130-3	MBL

Supplementary Table 2. The primer sequence for quantitative RT-PCR

**Gene symbol**		**5' to 3'**
**ALPL**	Forward	5'-CAAGGACATCGCATATCAGCTAA-3'
	Reverse	5'-CAGTTCTGTTCTTCGGGTACATGT-3'
**Axin2**	Forward	5'-AGTGAGACGCTCTCCCTCACCA-3'
	Reverse	5'-GAAACGCGCATAGGTTTGCTGGAC-3'
**Caprin2**	Forward	5'-GGCAGCCGGGGAGTCAC-3'
	Reverse	5'-GCTTCCAACTGGTCTGGGTT-3'
**Cdk1**	Forward	5'-ATTGTGTTTTGCCACTCCCG-3'
	Reverse	5'-ACAGCGTCACTACCTCGTGT-3'
**Cdon**	Forward	5’-CTGCACACACAAACTCCCTG-3’
	Reverse	5’-TTGGTTTTGGTGAAACACCTATTG-3’
**CD68**	Forward	5’-CTTCCCACAGGCAGCACAG-3’
	Reverse	5’-CTTCCCACAGGCAGCACAG-3’
**CD146**	Forward	5’-CGGGTGTGCCAGGAGAG-3’
	Reverse	5’-GGCGGTGCTCATATTCACCA-3’
**Ets1**	Forward	5'-AGAGCCAGTCGTGGTAAACTC-3'
	Reverse	5'-TGAAGGATGACTGGCTGCTC-3'
**Gapdh**	Forward	5’-GACATGCCGCCTGGAGAAAC-3’
	Reverse	5’-AGCCCAGGATGCCCTTTAGT-3’
**Runx2**	Forward	5’-CACCGACAGTCCCAACTTCCT-3’
	Reverse	5’-ACGGTAACCACAGTCCCATCTG-3’
**p16**	Forward	5'-CCCAACGCCCCGAACT-3'
	Reverse	5'-GCAGAAGAGCTGCTACGTGAA-3'
**p21**	Forward	5'-GTCAGGCTGGTCTGCCTCCG-3'
	Reverse	5'-CGGTCCCGTGGACAGTGAGCAG-3'
**Wif1**	Forward	5'-CCCGATGTATGAACGGTGGT-3'
	Reverse	5'-GGTGGTTGAGCAGTTTGCTTT-3'
**Wnt9a**	Forward	5'-GGCCCAAGCACACTACAAG-3'
	Reverse	5'-AGAAGAGATGGCGTAGAGGAAA-3'
**αSMA**	Forward	5′-CTGACAGAGGCACCACTGAA-3
	Reverse	5′-CATCTCCAGAGTCCAGCACA-3′
